# Examining Stress and Residual Symptoms in Remitted and Partially Remitted Depression Using a Wearable Electrodermal Activity Device: A Pilot Study

**DOI:** 10.1109/JTEHM.2022.3228483

**Published:** 2022-12-12

**Authors:** Aoife Whiston, Eric R. Igou, Dónal G. Fortune, Analog Devices Team, Maria Semkovska

**Affiliations:** Department of PsychologyUniversity of Limerick 8808 Limerick V94 T9PX Ireland; Department of PsychologyUniversity of Southern Denmark 6174 5230 Odense Denmark

**Keywords:** Depression, electrodermal activity, relapse, residual symptoms, stress, wearable devices

## Abstract

Consistent evidence suggests residual symptoms and stress are the most reliable predictors of relapse in remitted depression. Prevailing methodologies often do not enable continuous real-time sampling of stress. Thus, little is known about day-to-day interactions between residual symptoms and stress in remitted depression. In preparation for a full-scale trial, this study aimed to pilot a wrist-worn wearable electrodermal activity monitor: ADI (Analog Devices, Inc.) Study Watch for assessing interactions between physiological stress and residual depressive symptoms following depression remission. 13 individuals remitted from major depression completed baseline, daily diary, and post-daily diary assessments. Self-reported stress and residual symptoms were measured at baseline and post-daily diary. Diary assessments required participants to wear ADI’s Study Watch during waking hours and complete self-report questionnaires every evening over one week. Sleep problems, fatigue, energy loss, and agitation were the most frequently reported residual symptoms. Average skin conductance responses (SCRs) were 16.09 per-hour, with an average of 11.30 hours of wear time per-day. Increased residual symptoms were associated with enhanced self-reported stress on the same day. Increased SCRs on one day predicted increased residual symptoms on the next day. This study showed a wearable electrodermal activity device can be recommended for examining stress as a predictor of remitted depression. This study also provides preliminary work on relationships between residual symptoms and stress in remitted depression. Importantly, significant findings from the small sample of this pilot are preliminary with an aim to follow up with a 3-week full-scale study to draw conclusions about psychological processes explored. Clinical and Translational Impact Statemen—ADI’s wearable electrodermal activity device enables a continuous measure of physiological stress for identifying its interactions with residual depressive symptoms following remission. This novel procedure is promising for future studies.

## I. Introduction

Depression is the largest contributor to disability worldwide, affecting over 300 million individuals [1]. Much of the depression’s disease burden is linked to the disorder’s refractory nature and high relapse rates [2]. Depression relapse refers to the return of depressive symptoms after achieving remission, that is, not meeting the major depressive disorder (MDD) criteria for 2–3 weeks [3]. Following the first episode of depression, there is a 50-60% risk of future relapse; for subsequent episodes, the risk reaches 70-90% [4], [5], [6]. Previous research identifies both residual symptoms and stress as strong proximal predictors of this ‘relapse-remitting’ course of depression [7], [8], [9], [10], [11], [12], [13].

Residual symptoms are those which may persist, in milder forms, in individuals considered in remission following acute-phase treatment [14], [15]. Fatigue, feelings of guilt, depressed mood, anxiety, cognitive, and sleep problems are frequently reported residual symptoms, with 70-90% of individuals experiencing at least one such symptom post-treatment [16], [17], [18], [19]. These lingering symptoms increase relapse risk and impair psychological functioning [20], [21]. To understand relationships between residual symptoms and relapse, research also identified stress - the body’s non-specific response to any demand for change to play a central role [22]. Harkness et al. [23] demonstrated, 90% of the effect of residual symptomology on depression relapse was mediated by self-reported stress. Similarly, Morris et al. [24] identified increased residual symptomology in those exhibiting higher physiological reactivity to stressors. In line with previous stress research, both studies measured stress via retrospective self-report or one-time saliva sampling [25]. A limitation associated with these methodologies is that they do not unobtrusively enable the continuous real-time sampling of stress [25]. Thus, limited knowledge exists on the day-to-day interactions between residual symptomology and stress in remitted depression [26]. However, this type of research has become possible in the last decade due to advancements in smart wearable sensors.

Smart wearable sensors are wireless devices embedded in either garments (e.g., smart shirts) or accessories (e.g., wrist watches devices) capable of monitoring one or numerous bio-signals such as electrodermal activity (EDA), skin temperature (ST), electrocardiogram (ECG), and/or motion tracking (MT) [27]. Often, bio-signals measured via smart wearables, such as EDA and ECG, represent physiological changes within the body induced by stress hormones (cortisol, adrenaline, and noradrenaline), capable of providing a continuous measure of stress. Aside from ECG (heart rate signal), EDA (skin conductance signal) is one of the most prominent, unobtrusively recordable signals for the determination of stress using wearable sensors [25].

EDA is a measurement of the change in electrical properties of the skin resulting from stress or emotional arousal [28]. EDA signals consist of two components: the skin conductance level (SCL; slow-changing, underlying EDA signals) and the skin conductance response (SCR; faster changes in the EDA signal in response to external events and stressors) [29]. Smart wearable sensors detect minute changes in EDA signals by making use of an electrical current applied over electrodes that touch the skin [30]. Although EDA sensors do not feature in many commercially available wearables (e.g., Apple Watch), research-grade devices such as the ‘ADI Study Watch’, ‘Moodmetric Ring’, ‘Empatica E4’, and ‘iCalm’ have been empirically validated for EDA stress-detection in daily-life studies involving clinical and non-clinical samples [31], [32], [33], [34], [35], [36], [37], [38], [39], [40], [41].

To date, only two studies have applied wearable EDA devices (Empatica E4) to investigate stress in depressed samples in everyday life. Ghanderharioun et al. [42], in an eight-week prospective study, demonstrated EDA peaks, also known as skin conductance responses (SCRs), are more frequent and stronger in depressed samples. Similarly, Pedrelli et al. [43] showed across 31 participants that EDA is a predictive factor of depression severity. These findings reflect that of previous research investigating EDA and depression in laboratory-based studies [44], [45], [46]. However, to our knowledge, no study has applied a wearable EDA device to monitor stress in remitted depression in everyday life.

*Aims of the Current Study*

Thus, the aims of this pilot were to (1) test, in preparation for a full-scale study, the procedures and assessment methods for the evaluation of the Analog Devices (ADI) Study Watch EDA sensor as a stress monitor in remitted depression and (2) obtain preliminary results on the longitudinal relationship between residual depressive symptoms and stress in remitted depression.

## II. Methods

This prospective pilot study was approved by the Faculty of Education and Health Sciences Ethics Committee at the University of Limerick, and informed consent was obtained from all participants.

### A. Study Population

The total sample comprised 13 individuals remitted from major depressive episodes - 12 recommended for clinical pilot studies [47]. Participants were 18 years of age or older and had to meet the following *inclusion* criteria: (1) diagnosed as in remission from a primary diagnosis of MDD according to the fourth edition of the Diagnostic and Statistical Manual of Mental Disorders (DSM-IV) criteria [48] – confirmed by the Structured Clinical Interview for Depression (SCID) (2) scored ≤20 on the Beck’s Depression Inventory-II (BDI-II) [49], [50] (3) completed psychological (e.g., cognitive behavioural therapy; CBT), pharmacological antidepressant medications (ADM’s) or combined treatment (e.g., CBT plus ADM’s) for their previous depressive episode(s). ADM’s were permitted during the course of the study provided no recent (within 3-months) changes to dosage, or medication type, (4) can read and speak English fluently, (5) capable of following research procedures, (6) owner of a smartphone and (7) can provide informed consent. The *exclusion* criteria were: (1) a current diagnosis of a major depressive episode according to DSM-IV criteria [48], (2) a history of or current psychotic episode, according to the DSM-IV criteria [48], (3) significant visual problems and (4) pregnancy [51].

### B. Procedure

#### 1) Recruitment

In November 2020, we started recruiting participants at the University of Limerick. Due to restrictions during the COVID-19 pandemic, recruitment posters were distributed via email to the student population. Interested individuals contacted the researcher via email to receive a study information pack, which included a detailed information letter, research privacy notice, informed consent form, and a link to an online screening questionnaire. Individuals who returned completed consent forms, completed the online screening questionnaire, and met the inclusion criteria (1)-(7) were then invited forward for SCID assessment to confirm no current MDD episode.

#### 2) Screening

The study information packs included an information letter, research privacy notice, informed consent form, and a link to an online screening questionnaire. Participants were provided with the opportunity to ask any questions before completing the online screening questionnaire. Specific consent was sought for both the collection of daily diary data and the wearing of the ADI Study Watch for a one-week period should eligibility be confirmed. The online screening tool included questions on participant demographics (e.g., age and gender), previous depressive episode(s) (e.g., prior hospitalization and duration), and general health (e.g., other medical conditions and medications). Individuals returning consent forms, the online screening questionnaire, and meeting inclusion criteria (1)-(7) were invited forward for SCID diagnostic interview to confirm no current episode of MDD. The diagnostic interview was conducted by PhD researcher (author AW) supervised by a clinical psychologist (author DF). Due to COVID-19 restrictions in Ireland, all SCID interviews were conducted via telephone or Microsoft Teams at each participant’s discretion.

#### 3) Baseline and Daily Diary Assessments

For individuals eligible to enter the study based on their informed consent, screening questionnaire, BDI-II score, and SCID interview, their BDI-II assessment was used as a baseline assessment. Furthermore, the stress subscale of the Depression Anxiety and Stress Scale-21-S (DASS-21-S) was also administered to measure stress at baseline [52]. Eligible participants then began the daily diary and stress monitoring phase for one week. As well as wearing the ADI Study Watch during waking hours for a continuous measure of EDA, every evening, participants received an email with a link that directed them to a web-based diary questionnaire in a secure environment using Qualtrics XM. Daily diaries asked participants about their depressive symptomology and stress since waking up today using the BDI-II and the DASS-21-S. Daily diaries took approximately 10 minutes. Participants had a 3-hour window to fill in the diary questionnaire with a reminder delivered every hour. These diaries also asked participants to report any changes in medications or problems with procedures. Detailed information on the ADI Study Watch and daily diary measures are provided below. Engagement with study design, equipment, and procedures was also measured by identifying survey completion rates and average ADI Study Watch wear time.

#### 4) Exit Survey

For the exit survey, participants returned the ADI Study Watch and completed the BDI-II and DASS-21-S again. Furthermore, to assess the acceptability of the study design, procedures, and equipment, participants were asked questions relating to the feasibility of carrying out daily diary measures and wearing the ADI Study Watch for a 3-week period (full-scale study). Following assessments, participants were thanked for their participation, debriefed, and had the opportunity to ask further questions.

### C. Instruments

#### 1) Daily Diary Measures

Residual depression symptoms were measured using the BDI-II [49]. This 21-item measure required participants to self-report depressive symptom severity on a scale of 0–3 [49]. All BDI-II items are reflective of the DSM-IV criteria of MDD. Self-reported stress was measured using the DASS-21 stress subscale (DASS-21-S) [52]. This 7-item scale required participants to report levels of chronic non-specific arousal on a scale of 0-3. For this study, the BDI-II and DASS-21-S were altered by asking participants “rate how you have been feeling since waking up today”, compared to their original time-frames of the ‘past two weeks’ and ‘past week’ respectively [53], [54].

#### 2) ADI Study Watch

**FIGURE 1.**
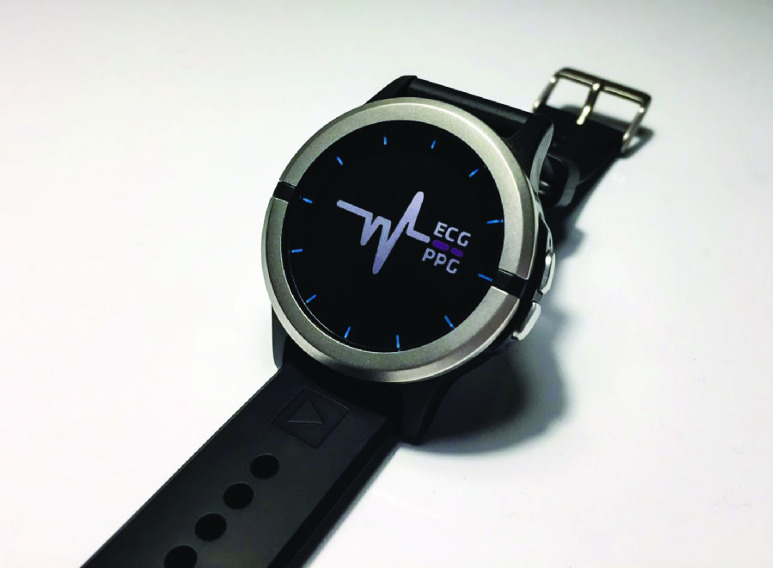
ADI study watch.

Developed by Analog Devices, the ADI Study Watch is a compact, battery-operated wrist-worn vital signs monitor (Figure 1). The ADI Study Watch includes technology simultaneously supporting; motion tracking, biopotential, optical heart rate, bio-impedance, and temperature measurements [55]. For this study, bio-impedance, more specifically EDA, was the measurement of interest, given that skin conductivity changes with increased stress. The ADI Study Watch detects minute changes in conductivity by using an alternating current (AC) excitation signal applied over two dry electrodes [55]. The ADI Study Watch applies an excitation signal of a maximum frequency of up to 200Hz. Importantly, the watch’s EDA sensors work on a low power consumption rate ( $ < 80 ~\mu \text{A}$ at an output rate of 4Hz) and are thus specifically designed for a continuous (36-48 hours) measurement of stress in a single charge [30]. All data from the ADI Study Watch devices were stored in flash memory retained on the device [55].

### D. Data Analysis

#### 1) ADI Study Watch Data Cleaning and Pre-Processing

EDA data from the ADI Study Watch was transferred via USB to ADI’s Vital Signs Monitoring (VSM) WaveTool program. From here, raw data logs were downloaded to a secure computer. Real, imaginary, magnitude, and phase angle components of both admittance and impedance signals are provided in the logs. The real part of admittance (conductance), provided in the unit of micro Siemens ( $\mu \text{S}$) sampled at 4Hz, was the measure used for the current analysis. This was necessary for use in the artifact and skin conductance response (SCR) detection algorithms outlined below. EDA data were then sorted into separate csv files representing daily intervals for each participant.

Each participant’s daily interval EDA files were then analyzed using the ‘EDA Explorer’ custom made algorithms for the Python-programming environment [56]. First, as EDA data was collected in ambulatory settings, noise/artifacts were likely present, signals were run through both a low-pass Butterworth Filter and EDA Explorer’s artifact detection algorithm. The low-pass Butterworth Filter removes line noise in the EDA signal. To apply this filter EDA Explorer up samples the data to 8Hz by linear interpolation and uses a sixth-order Butterworth low pass filter with a cut-off frequency 1Hz to filter the data. Regarding, the artefact detection algorithm, EDA Explorer was trained using a Support Vectors Machines (SVMs) machine learning binary classifier. First, two EDA experts judged 1,560 five-second EDA intervals as either clean or containing artifact. Both experts agreed on the following set of criteria defining an artifact: (a) EDA peaks that do not show exponential decay, (b) quantization error within >5% of signal amplitude, (c) sudden changes in EDA correlated with motion and (d) a SCL < 0. Labelled EDA data was then used as input for the machine learning binary classifier. After testing a variety of models, Radial Basis Functioning SVMs with the following parameter settings ( $\beta =0.1$, C=1000) showed greatest accuracy (95.67%). Thus, for the current study, the machine learning binary classifier automatically detected noise within EDA signals, labelling every five seconds of the signal as either ‘containing artifact’ (−1) or ‘containing no artifact’ (1).

Second, filtered EDA signals were processed to detect skin conductance responses (SCRs) and associated features including ‘EDA’ – the amplitude at the apex in $\mu \text{S}$, ‘Amp’ – amplitude of SCR, that is [amp = (EDA at apex) - (EDA at start of the SCR)] in $\mu \text{S}$, and ‘Max_deriv’ – maximum derivative of SCR in $\mu \text{S}$ per second. EDA Explorer’s SCR detection algorithm uses a through-to-peak (TTP) analysis whereby first- and second-order derivatives of the SCR signal are evaluated for speed, and acceleration changes are typically found when a SCR occurs. The current study adopts most of EDA Explorer’s default peak detection settings, that is, (a) maximum four second rise time – time it takes for an SCR to rise from the start of the SCR to the apex cannot exceed 4 seconds, (b) maximum four second decay time- time it takes for the SCR to decay to 50% of its amplitude cannot exceed four seconds, and (c) one second offset – the derivative must be positive for at least one second before a peak and negative for one second after a peak. However, we used a TTP threshold (or sensitivity) of $0.01~\mu \text{S}$ in the current study. This threshold was chosen as (a) it is most commonly used for modern EDA devices and (b) it has previously been applied and tested in EDA Explorer [29], [57], [58]. Python scripts and more information on EDA Explorer’s artefact and peak detection algorithms can be found here.

As SCRs are the measure of interest to the current analysis, to further ensure no artifacts were counted as SCRs, initially only SCRs in clean ‘containing no artifact’ EDA data were kept. However, as EDA Explorer’s artifact detection method classifies entire 5-second intervals as ‘containing artifact’, it is possible clean SCRs could have occurred in this time frame. Thus, SCRs within ‘containing artifact’ intervals were retained if they fell within two standard deviations with regards the ‘EDA’, ‘Amp’, and ‘Max_deriv’ of all ‘clean’ SCRs for each participant. SCRs were then incorporated into daily diary measures by calculating the average number of SCRs per-hour for each daily interval file for each participant. This was necessary due to participants wearing watches for different lengths of times per day.

Importantly, ‘EDA Explorer’ was chosen as the analysis tool for the current study as; (a) it has demonstrated to outperform other ‘gold standard’ EDA analysis programs for peak detection using their TTP method, (b) the freely available code is timesaving for handling large amounts of EDA data, and (c) the algorithms involved will enable us to avoid subjectivity in labeling peaks [29].

#### 2) Daily Diary Visualisations

All daily diary and ADI Study Watch data over the one-week daily assessment period were visualized at individual-level using code adapted from an experience sampling methodology visualisation programme’s ‘ESMvis’ plots [59]. ESMvis was chosen for the daily diary visualization tool for the current study as; (a) it is freely available, (b) easy to adjust to new datasets, and (c) enables the dynamic visualization of ESM time-series data [59].

#### 3) Multilevel Modelling (MLM)

To obtain preliminary results on the relationship between self-reported stress (DASS-21-S), physiological stress (SCRs), and residual depressive symptomology (BDI-II), two sets of multilevel models with level-1 effects were conducted. One set of MLMs examined the same-day relationships between the variables. The second set examined the lagged relationships, whereby predictors were brought down from time (T) to the row of data containing measurements at T+1. This was necessary to observe whether predictors at T predict the criterion variable(s) at T+1. Prior to running, all MLMs missing data were deleted listwise.

First, intercept-only (unconditional) models were conducted to check if residual depressive symptomology (criterion) shows significant between-participant variation and thus was suitable for MLMs. Second, SCRs and DASS-21-S scores (predictors) were participant mean-centred – subtracting each participant’s mean from each of their individual responses to remove between-participant variance. This was necessary as participants’ mean stress levels (e.g., average EDA) may differ meaningfully. Third, participant mean-centred predictors (SCR and DASS-21-S score) were entered into a random intercept/fixed slope model. This model suggests each participant has different mean levels of residual depressive symptomology, but the relationship between residual depression and stress is assumed to be similar across participants. Finally, participant mean-centred predictors were also entered in a random intercept /random slope model. This model suggests the relationship between residual depression and stress differs across participants. Furthermore, due to the exploratory nature of this feasibility study, MLMs were also run with residual depressive symptomology (BDI-II) as the predictor and stress- DASS-21-S and SCRs as separate criterion variables. All models followed the aforementioned procedure. MLMs were selected to analyse the daily diary aspect of the data due to their ability to model both within-participant and between-participants differences, and correcting for non-independence in the daily diary results.

## III. Results

### A. Sample Demographics

Thirteen participants (10 Female, 2 Male, and 1 Non-Binary) were recruited from the University of Limerick. Mean age was 25 (*SD* = 8, range 18-47). Five participants were actively taking antidepressant medications (Escitalopram, $n =3$; Fluoxetine, $n =1$; Vortioxetine, $n =1$). Seven participants reported one or more comorbid disorder(s) (General Anxiety Disorder, $n =6$; Social Anxiety Disorder, $n =1$; Panic Disorder, $n =1$; Sensory Processing Disorder, $n =1$). The average ( $\bar {\text {x}}$) BDI-II score at baseline across the sample was 10.23 (SD = 5.33; range 0 - 18) and at exit surveys was 5.88 (SD = 4.19; range 0 - 13). Five participants did not complete exit surveys. However, no significant difference in baseline BDI-II scores was observed between exit survey ‘completers’ ( $\bar {\text {x}} =8.25$, SD = 5.34) and ‘non-completers’ ( $\bar {\text {x}} =13.4$, SD = 3.85), t(10.63)= 2.02, p = 0.070. See Table 1 for a participant-level breakdown.

**TABLE 1:** Demographics Participant Level

	Participant Number												
	1	2	3	4	5	6	7	8	9	10	11	12	13
Gender	Non-Binary	Male	Female	Female	Male	Female	Female	Female	Female	Female	Female	Female	Female
Age	47	22	23	24	21	18	19	30	21	35	23	19	23
ADM	Yes	Yes	No	No	No	No	Yes	No	Yes	Yes	No	No	No
Comorbidities	No	Yes (GAD)	No	Yes (GAD)	Yes (GAD)	Yes (GAD,PD)	Yes (GAD, SPD)	No	NA	Yes (GAD)	Yes (SAD)	No	No
BDI-II Pre	14	13	2	10	16	13	12	5	0	9	13	8	18
BDI-II Post	13	NA	0	7	NA	9	NA	5	1	7	5	NA	NA
BDI-II Daily Assessment M(SD)	10.86 (7.29)	19.00 (11.40)	1.29 (1.80)	3.57 (3.21)	15.71 (3.40)	5.86 (5.96)	2.00(1.26)	3.57 (3.15)	2.29 (2.50)	4.86 (2.91)	4.29 (4.23)	4.86 (3.02)	3.14 (8.32)
Daily Assessments Complete	7	6	7	7	7	7	6	7	7	7	7	7	7

Note: GAD=general anxiety disorder. PD=panic disorder. SPD=sensory processing disorder. SAD=social anxiety disorder.

### B. Daily Diary Assessments

**FIGURE 2.**
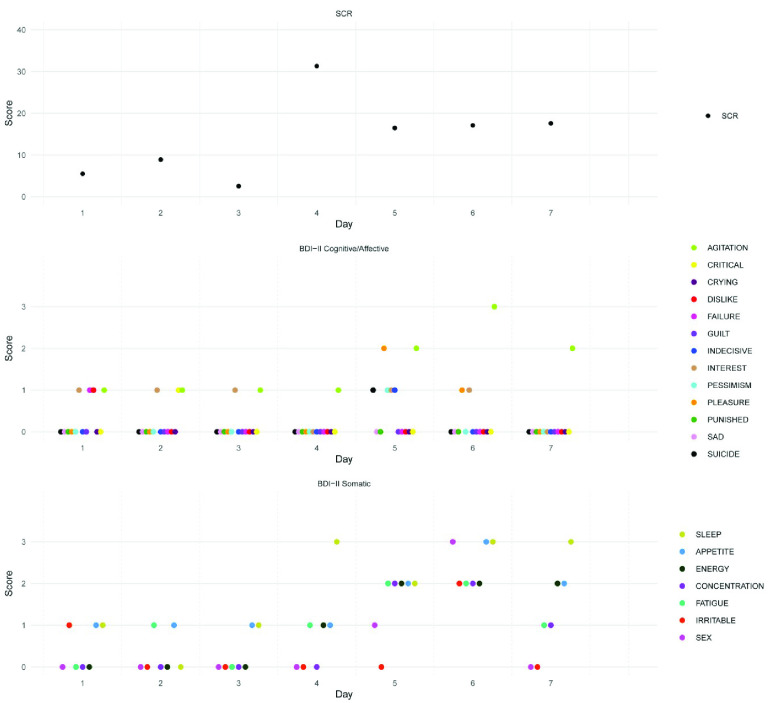
Sample average residual symptoms and SCR’s for participant 1.

Figure 2 displays one participant example (Participant 1) of ratings of residual depressive symptomology and average SCRs over the one-week daily diary period. However, due to the large variation in SCRs and residual symptomology between participants, it is recommended to view all other participant-level visualizations located in the supplementary materials (Supplementary Material 1-11).

#### 1) Residual Symptoms (BDI-II)

Regarding participant engagement over the 1-week daily diary period, there was a 97.80% response rate to residual symptom reports, with only two responses incomplete at Day 7. The most frequently reported residual depressive symptoms across all participants and surveys included *sleep problems* (absolute frequency; $f =48$), *fatigue* ( $f =38$), *energy loss* ( $f =33$), and *agitation* ( $f =30$). The least frequently reported residual symptoms included *suicide* ( $f =5$), *punished (* $f =5$), *crying* ( $f =7$) and *worthlessness (* $f =7$). For acceptability of diary procedures, of the eight participants completing exit surveys, all indicated it would be feasible to fill out these daily diaries for a 3-week full-scale study.

#### 2) ADI Study Watch Data

The following describes the group-level averages for ADI Study Watch data. See Table 2 for a participant-level breakdown. Watch data for one participant was not available. Thus all analyses, including ADI Study Watch data, are based on a sample of 12 participants.

**TABLE 2:** ADI Study Watch Feature Extraction Participant Level

Feature	Participant Number											
	1	2	3	4	5	6	7	8	9	10	11	12
Hours M(SD)	14.69 (1.06)	9.90 (2.39)	10.41 (1.73)	12.07 (2.60)	8.47 (1.79)	12.24 (2.33)	12.39 (4.68)	9.69 (2.27)	13.24 (2.69)	13.28 (1.63)	11.07 (2.87)	7.03 (1.87)
SCR M(SD)	14.21 (9.64)	41.9 (8.42)	17.41 (4.25)	5.08 (4.59)	13.7 (1.78)	14.31 (12.71)	6.28 (4.62)	13.61 (8.41)	22.34 (6.00)	7.54 (3.56)	6.21 (3.40)	26.92 (12.54)
EDA M(SD)	0.122 (0.082)	0.475 (0.102)	0.229 (0.108)	0.119 (0.092)	0.274 (0.152)	0.112 (0.033)	0.088 (0.019)	0.151 (0.108)	0.267 (0.089)	0.125 (0.047)	0.083 (0.021)	0.337 (0.175)
AMP M(SD)	0.024 (0.009)	0.062 (0.010)	0.046 (0.008)	0.024 (0.010)	0.032 (0.006)	0.023 (0.006)	0.018 (0.002)	0.036 (0.018)	0.059 (0.012)	0.038 (0.018)	0.026 (0.005)	0.048 (0.017)
Noise %	5.79 (7.08)	54.00 (16.01)	49.1 (17.71)	17.75 (21.45)	15.03 (20.31)	10.74 (14.37)	1.67 (0.97)	24.26 (22.32)	45.04 (19.87)	10.17 (5.48)	22.55 (7.65)	47.31 (36.91)

Note: SCR=skin conductance response. EDA = the amplitude of SCR’s at the apex in $\mu$ S. AMP = amplitude of SCR, that is [amp =(EDA at apex) - (EDA at start of the SCR)] in $\mu$ S. Participant 13 is omitted due to ADI Study Watch data not available.

For ADI Study Watch engagement, the average wear time was 11.30 hours per day (*SD* = 3.09; range 3.41-17.45). Eight of 84 days of watch data were missing across the sample, often due to user technical issues (e.g., letting the battery run out and changing sensor settings). The average number of SCRs was 16.09 per-hour (*SD* = 12.37; range 0.87 - 52.19). SCRs had an average EDA of $0.197 ~\mu \text{S}$ (*SD* = 0.145; range 0.009- 0.566) and Amp of $0.037 ~\mu \text{S}$ (*SD* = 0.018, range 0.013- 0.0743). Although only clean SCRs were kept for analysis, the percentage of noise/artifacts in EDA data also varied considerably. The average percentage of noise per hour across the sample was 26.16 % (SD = 24.83%; range 0.26- 94.71%). For acceptability of procedures, of the eight participants completing exit surveys, all indicated that wearing the ADI Study Watch would be feasible for a 3-week full-scale study.

#### 3) Multilevel Modelling

See Table 3 for the results of all multilevel analyses with stress (DASS-21-S and SCRs) as predictors and BDI-II as a criterion. The intercept-only model was significant, suggesting 58% of the variance in BDI-II scores is due to participant-to-participant variation and thus suitable for MLM.

**TABLE 3:** Multilevel Models With BDI-II as Outcome, DASS-21-S and SCR as Predictor

BDI-II as Outcome															
Unconditional Model			Same-Day RE/FS			Same-Day RE/RS			Lag-Day RE/FS			Lag-Day RE/RS			
Predictors	Estimates	CI	p	Estimates	CI	p	Estimates	CI	p	Estimates	CI	p	Estimates	CI	p
(Intercept)	6.61	3.24 – 9.97	< 0.001	6.62	3.24 – 10.01	< 0.001	6.65	3.24 – 10.06	< 0.001	5.89	2.75 – 9.04	< 0.001	5.83	2.98 – 8.69	< 0.001
SCR				0.05	−0.08 – 0.18	0.425	0.13	−0.13 – 0.38	0.322						
DASS-21-S				1.13	0.71 – 1.54	< 0.001	0.80	0.34 – 1.27	0.001						
SCR_lag										0.19	0.03 – 0.35	0.023	0.22	−0.06 – 0.49	0.127
DASS-21-S_lag										0.29	−0.19 – 0.78	0.231	0.05	−0.58 – 0.68	0.870
Random Effects															
$\sigma 2$	23.24			15.98			6.91			20.29			10.29		
$\tau 00$	31.62 Subject			33.17 Subject			35.18 Subject			26.79 Subject			23.45 Subject		
$\tau 11$							0.17 Subject.SCR						0.19 Subject.SCR_lag		
							0.30 Subject.DASS-21-S						0.56 Subject.DASS-21-S_lag		
$\rho 01$							0.92						1.00		
							0.48						0.71		
ICC	0.58			0.67			0.84			0.57					
N	12 Subject			12 Subject			12 Subject			12 Subject			12 Subject		
Observations	75			75			75			63			63		
Marginal R2 / Conditional R2	0.000 / 0.576			0.118 / 0.713			0.095 / 0.852			0.048 / 0.590			0.187 / NA		

Note: $\sigma^{2}$ = Within-person residual variance. $\tau_{00}$ = Between-day variance, $\tau_{ll}$ = Between-person variance. ICC = proportion of variance explained by between-person differences.

For both the random effects/fixed slope and random effects/random slope models, the same day relationship between DASS-21-S and BDI-II scores was significant. On days participants self-reported greater BDI-II scores, their self-reported DASS-21-S scores were also higher. However, for the random effects/random slope model, a singular fit was obtained, indicating this model’s structure is too complex to be supported by our data. There was no significant same-day relationship between SCR and BDI-II scores across both models.

For the random effects/fixed slope model, the lagged relationship between SCR and BDI-II scores was significant. Greater SCRs on day T-1 was associated with increased BDI-II scores on day T. The random effects/random slope model did not demonstrate the same relationship. Again, the random effects/random slope model obtained a singular fit, indicating this model’s structure is too complex to be supported by the data. Furthermore, no significant lagged effects were shown with DASS-21-S scores as predictor and BDI-II as the criterion.

See Table 4 and 5 for the results of all multilevel analyses with the BDI-II as predictor and DASS-21-S and SCRs as criterion variables modelled separately. Intercept-only models were significant, suggesting 63% of the variance in SCRs and 56% of the variance in DASS-21-S scores is due to participant variation and thus suitable for MLM.

**TABLE 4:** Multilevel Models With DASS-21-S as Outcome and BDI-II Predictor

DASS-21-S Outcome																	
			Unconditional Model			Same-Day RE/FS			Same-Day RE/RS			Lag-Day RE/FS			Lag-Day RE/RS		
Predictors			Estimates	CI	p	Estimates	CI	p	Estimates	CI	p	Estimates	CI	p	Estimates	CI	p
(Intercept)			3.10	1.46 – 4.73	< 0.001	3.11	1.46 – 4.76	< 0.001	3.11	1.46 – 4.76	< 0.001	2.61	1.03 – 4.20	0.001	2.60	1.03 – 4.18	0.001
BDI-II						0.28	0.18 – 0.39	< 0.001	0.29	0.09 – 0.50	0.006				
BDI-II_lag												0.14	0.04 – 0.25	0.008	0.10	−0.05 – 0.24	0.190
Random Effects															
$\sigma 2$			5.79			3.95			3.29			3.00			2.55
$\tau 00$			7.36 Subject			7.83 Subject			7.99 Subject			7.25 Subject			7.24 Subject
$\tau 11$									0.07 Subject.BDI-II						0.03 Subject.BDI-II_lag		
$\rho 01$									0.73 Subject						0.91 Subject		
ICC			0.56			0.66			0.74			0.71			0.76
N			12 Subject			12 Subject			12 Subject			12 Subject			12 Subject
Observations			75			75			75			63			63
Marginal R2 / Conditional R2			0.000 / 0.560			0.120 / 0.705			0.119 / 0.772			0.034 / 0.717			0.016 / 0.761

Note: $\sigma^{2}$ = Within-person residual variance. $\tau_{00}$ = Between-day variance. $\tau_{II}$ Between-person variance. ICC = proportion of variance explained by between-person differences.

**TABLE 5:** Multilevel Models With SCR as Outcome and BDI-II Predictor

SCR as Outcome															
Unconditional Model			Same-Day RE/FS			Same-Day RE/RS			Lag-Day RE/FS			Lag-Day RE/RS			
Predictors	Estimates	CI	p	Estimates	CI	p	Estimates	CI	p	Estimates	CI	p	Estimates	CI	p
(Intercept)	15.83	9.85-21.81	<0.001	15.83	9.85-21.81	<0.001	15.82	9.84-21.81	<0.001	16.06	10.21-21.90	<0.001	15.89	10.39-21.38	<0.001
BDI-II				0.25	−0.15 – 0.65	0.222	−0.11	−0.77 – 0.56	0.752						
BDI-II_lag										0.28	−0.23 – 0.79	0.280	0.06	−0.54 – 0.67	0.844
Random Effects															
$\sigma 2$	60.73			60.26			52.59			68.96			64.00		
$\tau 00$	101.70 Subject			101.78 Subject			103.11 Subject			92.85 Subject			81.61 Subject		
$\tau 11$							0.48 Subject.BDI_II						0.26 Subject.BDI-II_lag		
$\rho 01$							0.16 Subject						0.89 Subject		
ICC	0.63			0.63			0.68			0.57			0.58		
N	12 Subject			12 Subject			12 Subject			12 Subject			12 Subject		
Observations	75			75			75			63			63		
Marginal R2 / Conditional R2	0.000 / 0.626			0.007 / 0.631			0.001 / 0.682			0.008 / 0.577			0.000 / 0.580		

Note: $\sigma 2=$ Within-person residual variance. $\tau 00=$ Between-day variance. $\tau 11$ = Between-person variance. ICC = proportion of variance explained by between-person differences.

For both the random effects/fixed slope and random effects/random slope models, again, the same day relationship between DASS-21-S and BDI-II scores was significant. On days participants self-reported greater DASS-21-S scores, their self-reported BDI-II scores were also higher. There was no significant same-day relationship between BDI-II and SCRs across both models.

For the random effects/fixed slope model, the lagged relationship between BDI-II and DASS-21-S scores was significant. Greater BDI-II scores on day T-1 were associated with increased DASS-21-S scores on day T. The random effects/random slope model did not demonstrate the same relationship. Furthermore, no significant lagged effects were shown with BDI-II as the predictor and SCRs as the outcome variable.

#### 4) Post-Hoc Multilevel Models

A final set of multilevel models was run with SCR as predictor and DASS-21-S as the criterion variable. This was conducted as (a) the same day relationship for BDI-II and DASS-21-S scores were significant and (b) the random effects/fixed slope lagged model showed SCR significantly predicted BDI-II scores. Thus, we wanted to examine if SCRs predicted DASS-21-S scores, particularly in the lagged models. No significant same-day or lagged effects were shown with SCR as predictor and DASS-21-S as the criterion.

## IV. Discussion

The pilot aimed to test the procedures and methods in preparation of a full-scale study of the use of a wearable electrodermal activity (EDA) monitor, the ADI Study Watch in remitted depression, and to obtain preliminary data on the relationships between residual depressive symptoms, physiological stress and self-reported over one week. Both the ADI Study Watch and daily diary measures showed high participant engagement and acceptability. Participants supported the use of materials and procedures for future use in a three-week full-scale study. Regarding residual depressive symptoms, *sleep problems*, *fatigue*, *energy loss*, and *agitation* were the most frequently reported. For the ADI Study Watch EDA sensors, the average number of skin conductance responses (SCRs) was 16.09 per hour with an average daily wear time of 11 hours. Regarding preliminary relationships between residual depressive symptoms and stress, self-reported stress was also higher on days where participants self-reported greater residual depressive symptoms. For physiological stress, increased SCRs on one day predicted an increase in reports of residual depressive symptoms on the next day.

Daily diary measures showed *sleep problems*, *fatigue*, *energy loss,* and *agitation* were the most frequently reported residual symptoms. This result is consistent with previous research showing 70-90% of individuals experience at least one residual symptom post-treatment [16], [17], [18], [19]. Moreover, although somatic symptoms -*sleep problems, energy loss,* and *fatigue* have previously been shown to be common residual symptoms post-treatment, particularly in student samples, *agitation* has not shown a similar prevalence [60], [61]. Greater reporting of *agitation* in the current study may relate to half the current sample also reporting previous comorbid anxiety disorders, with *agitation* showing to be a key bridge (comorbidity developing and maintenance) symptom between depression and anxiety disorders [62]. Similarly, network analysis shows anxiety-related symptoms (e.g., agitation) from distinct symptom clusters together with somatic symptoms [63]. Identifying residual somatic and agitation symptoms is important as if not relieved or regulated, these symptoms often show higher relapse risk and a more chronic course of depression [63], [64].

Moreover, daily diary measures showed high levels of engagement and acceptability, with participants completing the majority of assessments and reporting measures would be feasible to answer in a 3-week full-scale study. Nevertheless, limitations existed with the current diary measure. Administering the 21-item BDI-II on a small sample over a 1-week period resulted in insufficient power to map complex interactions that may exist between specific residual symptoms and stress post-treatment [65]. The full-scale study will use a shorter daily diary measure (e.g., PHQ-9), and increase the sampling frequency (twice daily- AM & PM). This will enable a shift from simply listing and counting residual symptoms towards investigating their differences in risk factors, interactions, and consequences via network psychometrics [65], [66].

This study also showed it is feasible to use ADIs’ Study Watch for EDA measurement over a 1-week daily assessment phase. Similar to previous research, the compact, wrist-worn, dry electrodes were easily applied, re-usable, and readily tolerated for prolonged continuous wear (24 + hours) [67]. Moreover, as the ADI Study Watch has customizable sampling rates providing raw data outputs on real, imaginary, magnitude, and phase angle components of EDA signals; this device can be easily adapted to work alongside previously developed artefact and peak detection algorithms, such as EDA Explorer [30].

Regarding the ADI watch sensor signals, current results show average EDA and amplitude of the SCRs were relatively low. This lower signal is expected and may relate to monitor placement, with wrist-worn dry electrodes situated further from palmer sites [32]. Nevertheless, research shows that, although lower absolute signal levels are observed from the wrist, SCRs measures vary in a similar fashion to palmer site electrodes [68]. Thus, while this should not affect the overall relationships presented here, it may affect the strength of relationships. In fact, if the ADI Study Watch EDA sensors were closer to palmer sites, we may expect stronger lagged relationships between SCRs and residual depressive symptoms.

For signal artifacts, percentages varied considerably with some participants showing very-low artifact percentages (e.g. 1.67%, 5.79%), others very-high (e.g. 36.91%, 22.32%) even after filtering the data. This result reflects previous research showing in unrestricted daily-life environments 10% (range, 6-24%) of EDA signals contain artifacts either arising from electrode-skin interface obstruction, external electrode pressure, and/or re-adjusting electrode position [69], [70]. Specific to this study, greater signal artifacts may also relate to the non-contact procedures required during a national COVID-19 lockdown. As researchers could not physically check optimal/correct watch placement, incorrect placements may have occurred, potentially reducing signal quality. Greater emphasis and education of importance of correct monitor use and placement will be provided in the full-scale study either in person or via brief video demonstrations.

This study also obtained preliminary results on longitudinal interactions between residual depressive symptoms and stress- physiological (SCRs) and self-reported (DASS-21-S). For physiological stress, increased SCRs on one day were associated with increased residual symptoms on the next day. This result in consistent with previous research on current a depressive episode(s) showing SCRs predict increase in depression severity [42], [43]. Explanations for this possibly lay with the physiology of the stress response and somatic depressive symptoms. The stress response, indirectly measured by SCRs, is a complex mechanism promoted by the sympathetic nervous system, hypothalamic pituitary adrenal axis (HPA), and the release of stress hormones- cortisol, adrenaline, and noradrenaline [25]. Research shows, HPA axis hyperactivity and elevated cortisol often lead to depressive symptoms, particularly somatic symptoms– fatigue, sleep problems, and loss of energy [71], [72]. Whilst this study could not map specific symptom-stress interactions, *fatigue*, *sleep problems,* and *loss of energy* were the most frequently reported residual symptoms, potentially contributing to the current result. Identifying this interaction is important as elevated SCRs may be a reliable biomarker for the development of residual somatic symptoms in remitted depression. Thus, stress management techniques could be a strategy, perhaps as part of a suite of relapse prevention interventions to reduce stress, potentially reducing the occurrence of somatic symptoms, and thus reducing the risk of depression relapse.

Regarding self-reported stress, higher self-reported stress ratings on one day were associated with high residual symptom ratings on the same day and vice-versa. One possible explanation for this result may relate to the psychometric properties of the DASS-21 stress (DASS-21-S) subscale and the sample characteristics. Originally developed to measure anxiety and depression, testing of the DASS-21-S revealed a third factor, namely stress [52]. Since then, mixed results have emerged for this scale’s three-factor structure [73]. For example, previous research shows the stress subscale, measuring persistent states of arousal, greatly overlaps with anxiety symptoms leaving divisions between the two subscales arbitrary [52], [62]. Moreover, somatic symptoms- loss of energy, sleep, and appetite problems, have also shown to be closely related to the DASS-21-S subscale [52]. Thus, the same-day relationship between residual symptoms and self-reported stress may relate to half of the current sample reporting comorbid anxiety disorders and/or the higher frequency of somatic symptom reporting. Alternatively, this result may also be related to the maintenance of cognitive biases in remitted depression. For example, a recent meta-analysis [74] showed persisting memory biases towards negatively-valenced self-referencing words in remitted individuals. Thus, they are likely to score high on both negatively valenced self-reported residual symptom and stress measures when these are administered simultaneously.

It is also important to note, physiological stress (SCRs) did not predict self-reported stress scores (DASS-21-S) on either the same day or at the next day. While this conflicts with research identifying associations between self-report and physiological stress measures, it is not spurious [39]. For example, both Gidlow et al. [75] and Staufenbiel et al. [76] show self-reported stress is often inconsistent with behavioral and physiological measures. Explanations for this finding often center around lagged effects caused by different timing of the stress responses [75], [76]. While this explanation is possible, considering both the same-day relationship between BDI-II and DASS-21-S scores and the lagged relationships with SCRs predicting increased BDI-II scores, conclusions drawn on relationships between SCRs and DASS-21-S scores should be interpreted with caution. Indeed, future research should seek to investigate this finding further.

### A. Limitations

Aside from the limitations discussed above, results should also be interpreted in light of the following. First, the small sample of a pilot study does not permit the analysis of complex relationships between specific residual depressive symptoms and stress nor the assessment of demographic (e.g., gender), clinical (e.g., comorbidities), and/or treatment (e.g., ADM’s) effects on the results. Whilst sample size calculations are not mandatory for pilot studies, our sample meets clinical pilot guidelines and thus adequate for the purpose of the study: ensuring the ADI Study Watch is useful for examinations stress and residual symptoms [47], [77]. Conclusions about the psychological processes measured cannot be drawn with certainty.

Second, recruitment to this study was via snowball sampling through university networks. Resultantly, the majority of the sample were students and female, meaning generalizations should be made with care. Importantly, for gender, our distribution reflects that of previous meta-analyses showing depression rates are 63% females and 37% males (Supplementary Material 12) [78], [79]. Nevertheless, subsequent studies may need to adjust recruitment strategies to obtain more balanced and diverse sample.

Third, half of the sample reported comorbid anxiety disorders, thus, potentially influencing the types of residual symptoms and results presented. Of the six reporting comorbid anxiety, 4 scored in the no anxiety symptom to normal range, 1 scored in the moderate range, and only 1 scored in the high anxiety symptom range [52]. This distribution was similar to participants reporting no comorbidities, 4 scoring in no-normal range, 1 in the mild range, and 2 in the moderate symptom range. Often, this is due to factors associated with remission also being associated with general psychological functioning. Resultantly, remission of one disorder - e.g., depression is likely to produce remission from another - e.g., anxiety [80].Thus, it is unlikely past or current comorbidities affected current results. Moreover, although excluding comorbidities is common practice in randomized controlled trials, it is not a true representation of depression in the real world [81]. Future studies should continue allowing comorbid disorders, which represent depression reality. This will enable comprehensive examinations of relationships between residual symptoms and stress.

Fourth, large heterogeneity was observed across the study’s participants in the reporting of residual depressive symptoms and measurement of physiological stress. The full-scale study will aim to conduct some idiographic-type analyses to explore how time-series of relationships between residual symptoms and stress in remitted depression differ across individuals [82].

Fifth, almost half ( $N =$ five) of the participants did not complete exit surveys. In most cases, exit surveys were administered after ADI Study Watch collection (e.g., watch collected in the AM after day seven). It is possible participants may have felt the study was complete once no longer wearing the watch and therefore not motivated to complete exit surveys. Although this was not investigated directly, greater emphasis will be placed on exit survey completion for the full-scale study.

Sixth, EDA Explorers’ machine learning binary classifier was used to detect noise within ADI Study Watch data, labeling every five seconds as ‘containing artifact’ or ‘containing no artifact’ [56]. Although EDA Explorers’ peak detection algorithm proves accurate in artifact-rich datasets, the same has not been shown for the artifact detection algorithm. Often, artifacts are overestimated in artifact-rich environments [29]. While this should not affect the relationships between residual symptoms and stress presented here, it may affect artifact percentages reported throughout. Moreover, EDA Explorer does not automatically account for simultaneous occurrence of artifacts and clean SCRs within the same five-second epochs. However, to account for this, the current study kept SCRs within ‘containing artifact’ intervals if they fell within two standard deviations with regards the ‘EDA’, ‘Amp’, and ‘Max_deriv’ of all ‘clean’ SCRs for each participant.

Finally, we add that lagged multilevel models represent Granger causality as SCRs precede residual depressive symptoms and SCRs are related to residual depressive symptoms [83]. This should not be interpreted as direct causality as many unknowns remain. For example, did we measure variables at the correct time frame, or did we capture residual symptoms and stress with reliable and valid measures free from bias [84] ? Thus, while this study should be interpreted as exploratory work, its value lies in generating hypotheses on interactions between residual symptoms and stress in remitted depression with an aim to follow up in a 3-week full-scale study.

## V. Conclusion

This pilot study was the first to use a wearable, wrist-worn electrodermal activity (EDA) monitor to examine stress and residual symptoms in remitted and partially remitted depression in everyday life. This study also provides preliminary results on relationships between residual symptoms and stress. Importantly, residual symptoms, particularly somatic, prevail in remitted depression. These symptoms are reported in greater amounts the day after greater physiological stress. Thus, elevated physiological stress, assessed by using the ADI Study Watch, could be a potential biomarker for residual symptomology. However, significant findings from pilot studies are preliminary and call for further examination to draw conclusions about the psychological processes that we explored. To further examine these relationships, we designed a 3-week full-scale study. Utilizing a larger sample, the ADI Study Watch, and a shorter but more frequent dairy measure, we hope to further disentangle the complex relationships between specific residual symptoms and stress in remitted depression. In essence, based on our current examination, we can recommend using this wearable device to examine stress as a predictor of remitted and partially remitted depression.

## Supplementary Materials

Supplementary materials
