# Supplementary material for: Examining Stress and Residual Symptoms in Remitted and Partially Remitted Depression Using a Wearable Electrodermal Activity Device: A Pilot Study
Source: IEEE J Transl Eng Health Med. 2022 Dec 12;11:96–106. doi: 10.1109/JTEHM.2022.3228483 (PMC9833495; doi:10.1109/JTEHM.2022.3228483)
Supplement: Supplementary materials [file supp1-3228483.pdf]

Supplementary Material 1: Participant 2

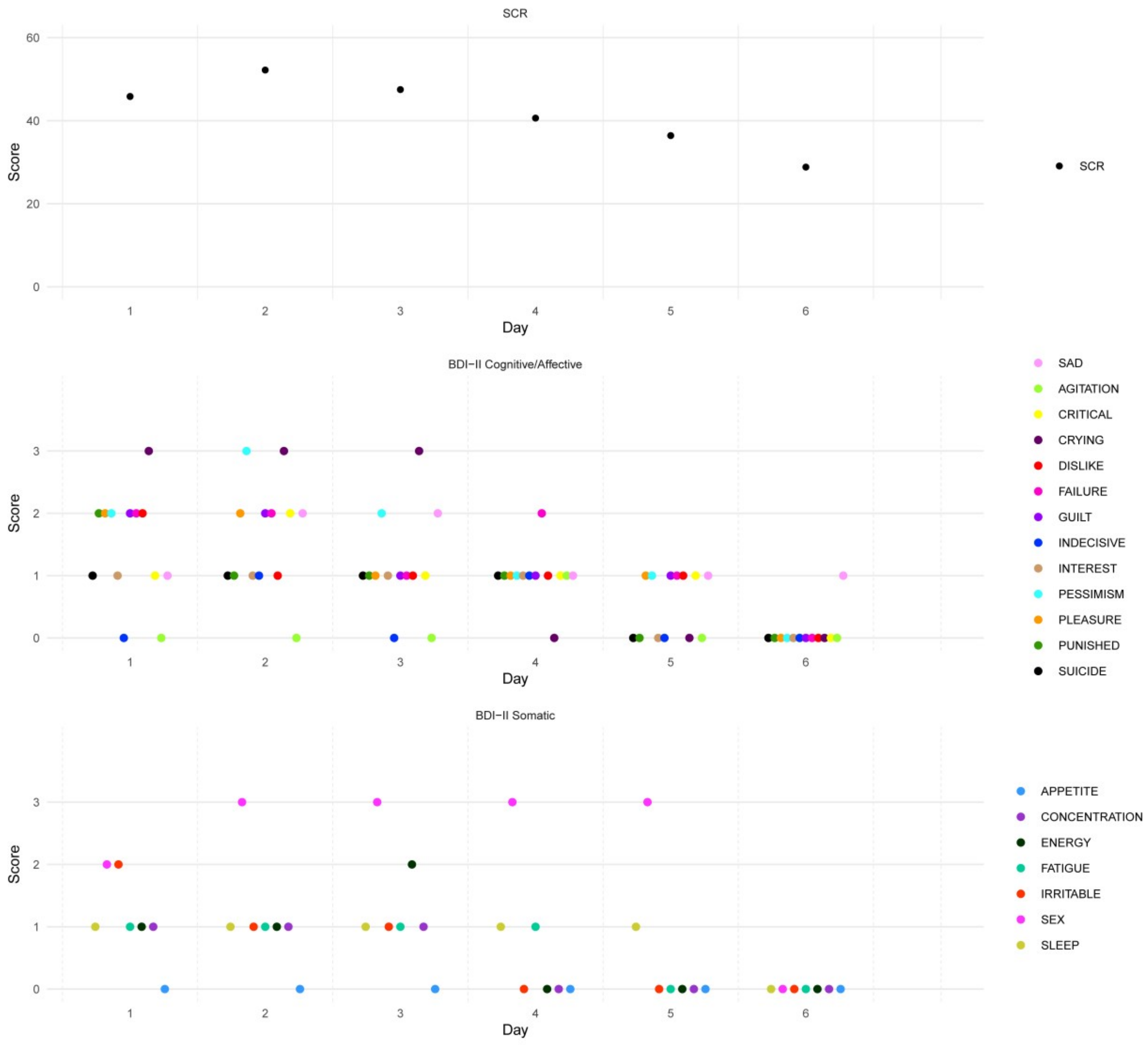

Supplementary Material 2: Participant 3

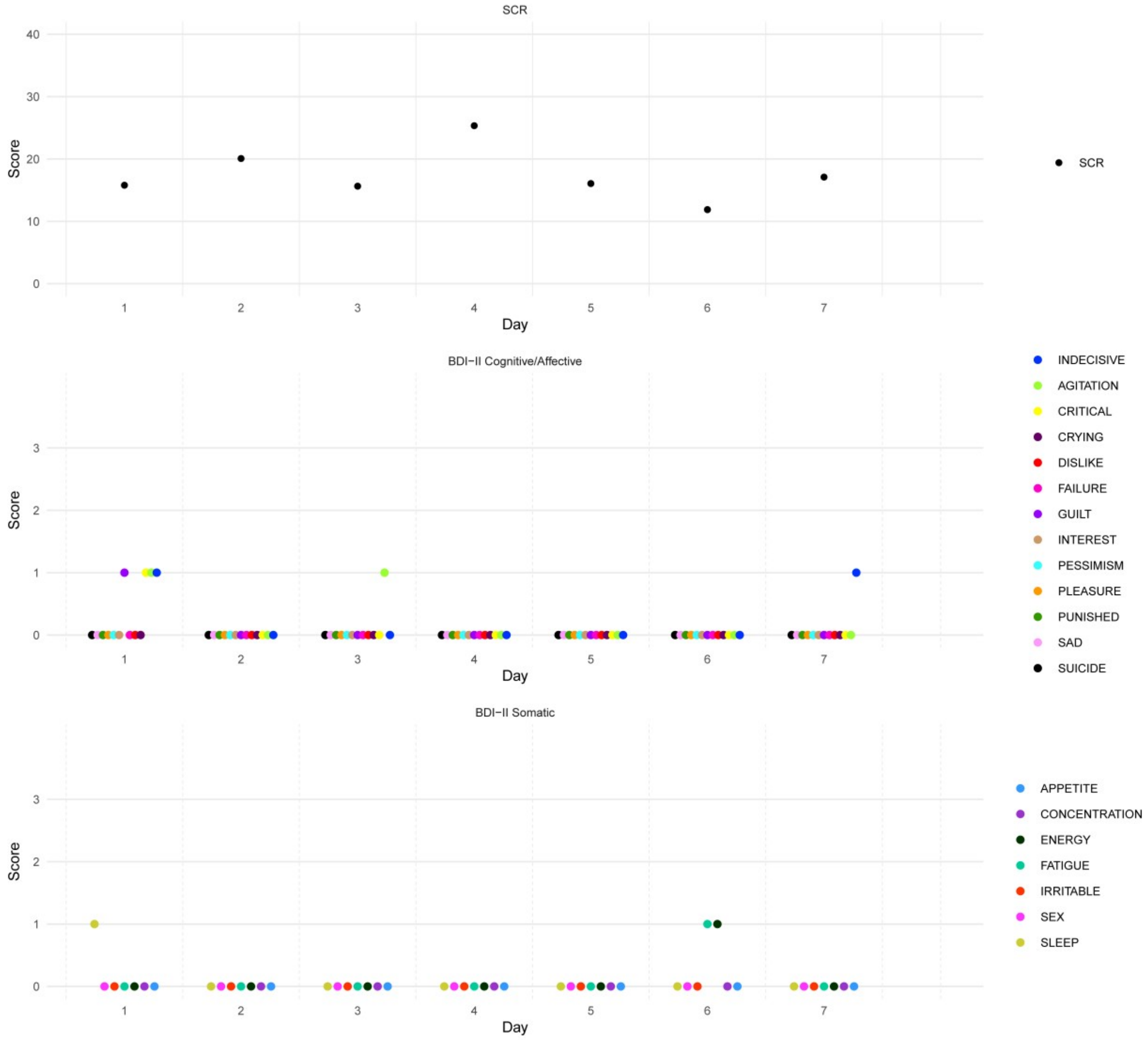

Supplementary Material 3: Participant 4

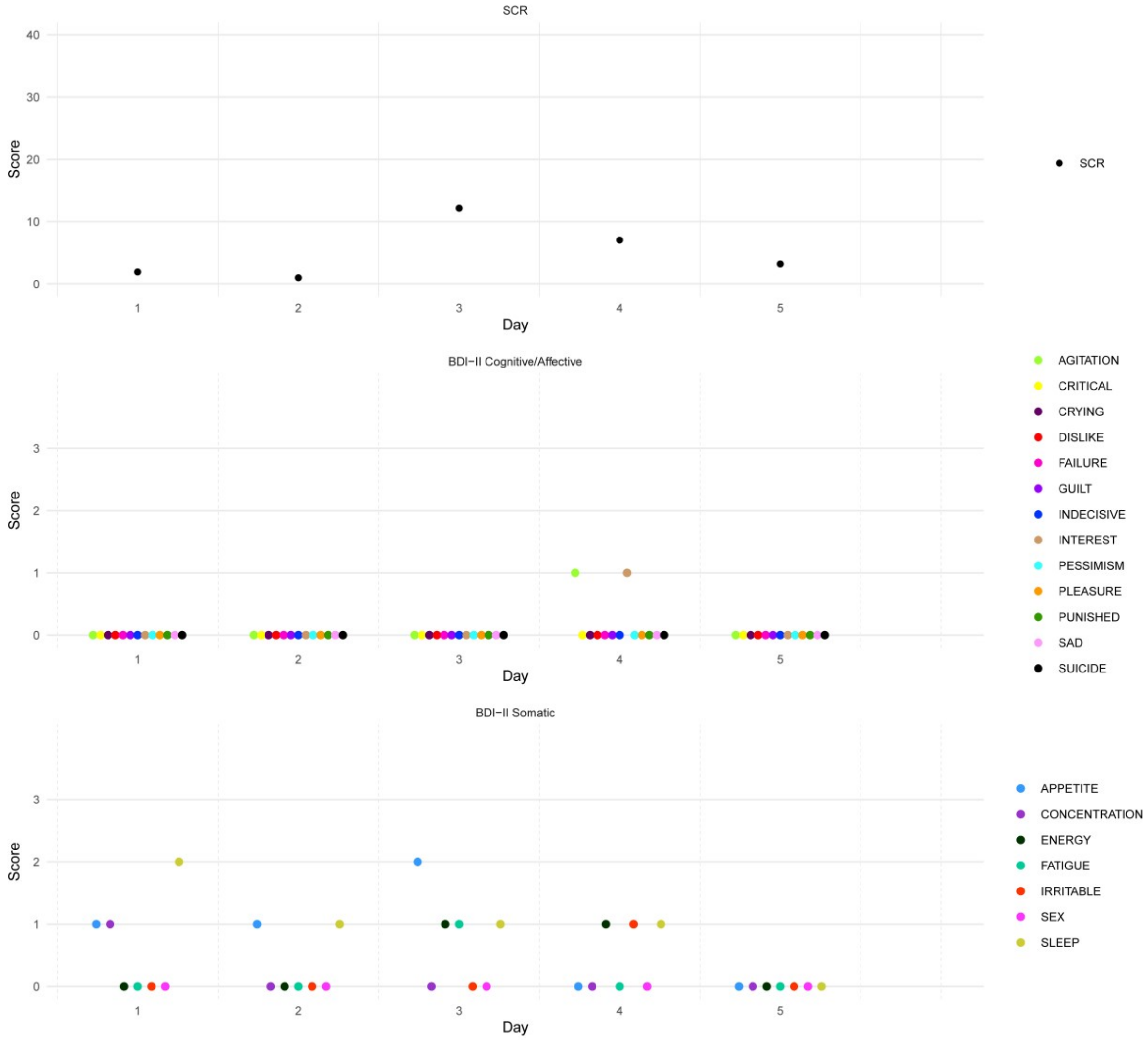

Supplementary Material 4: Participant 5

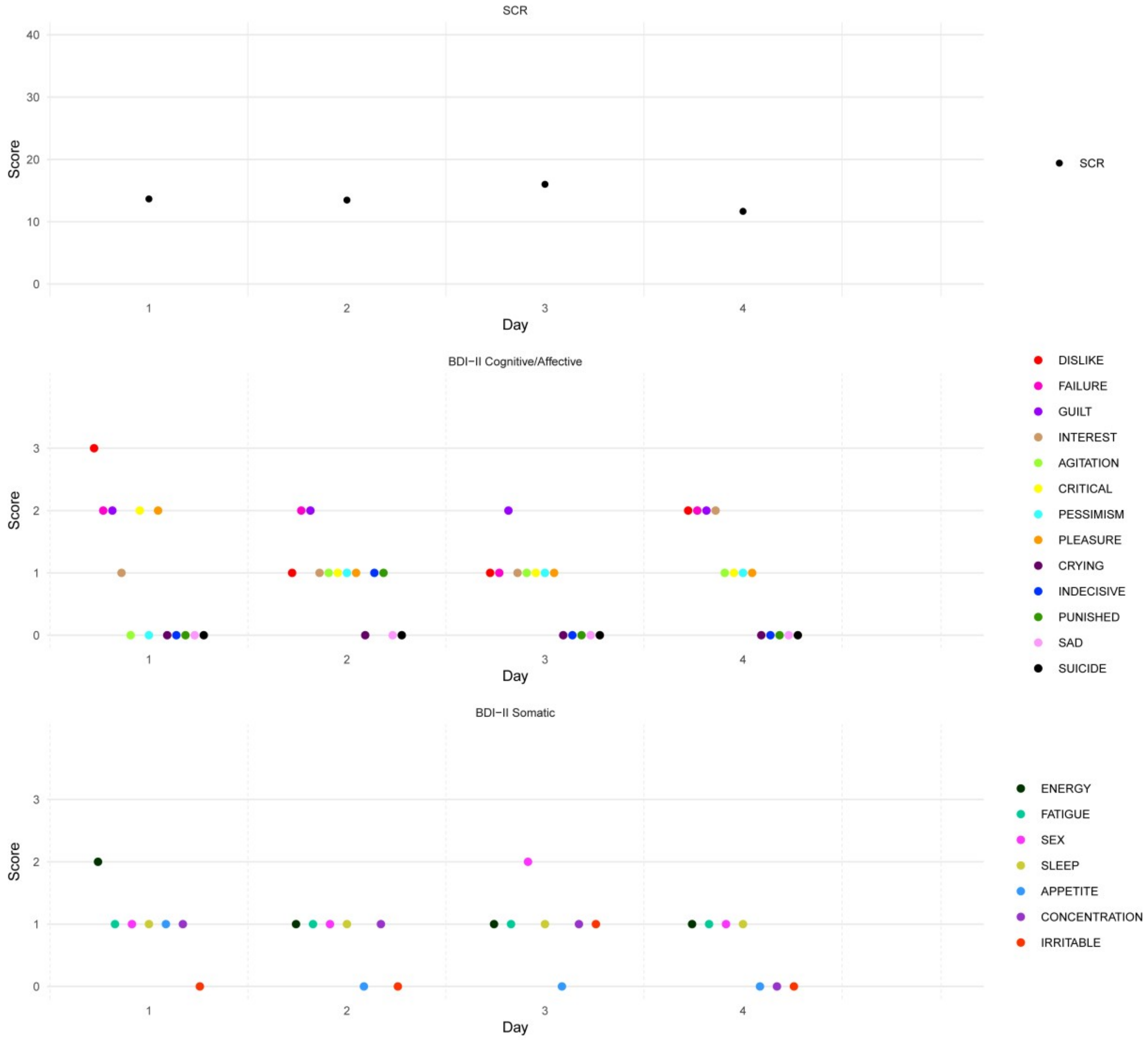

Supplementary Material 5: Participant 6

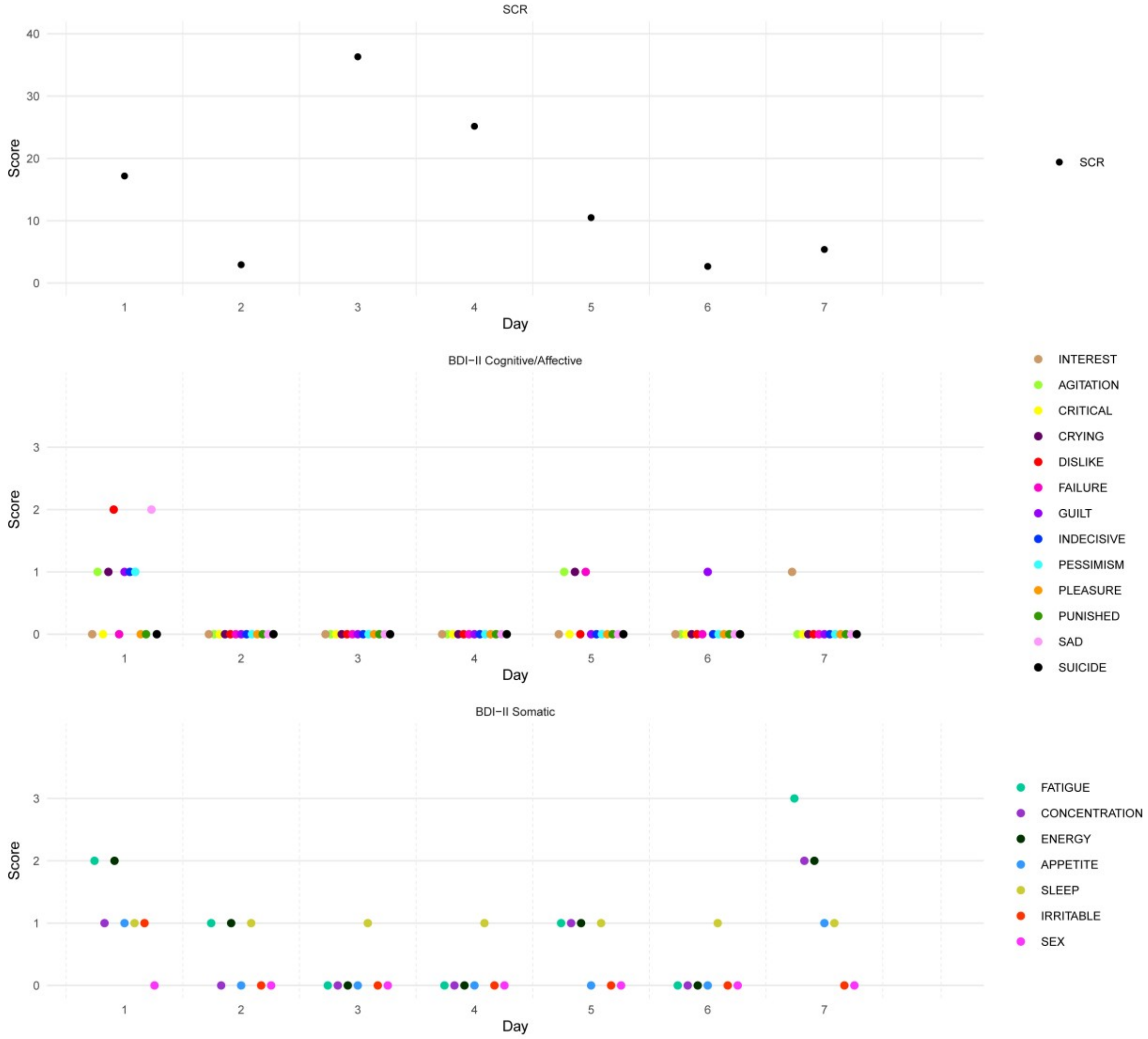

Supplementary Material 6: Participant 7

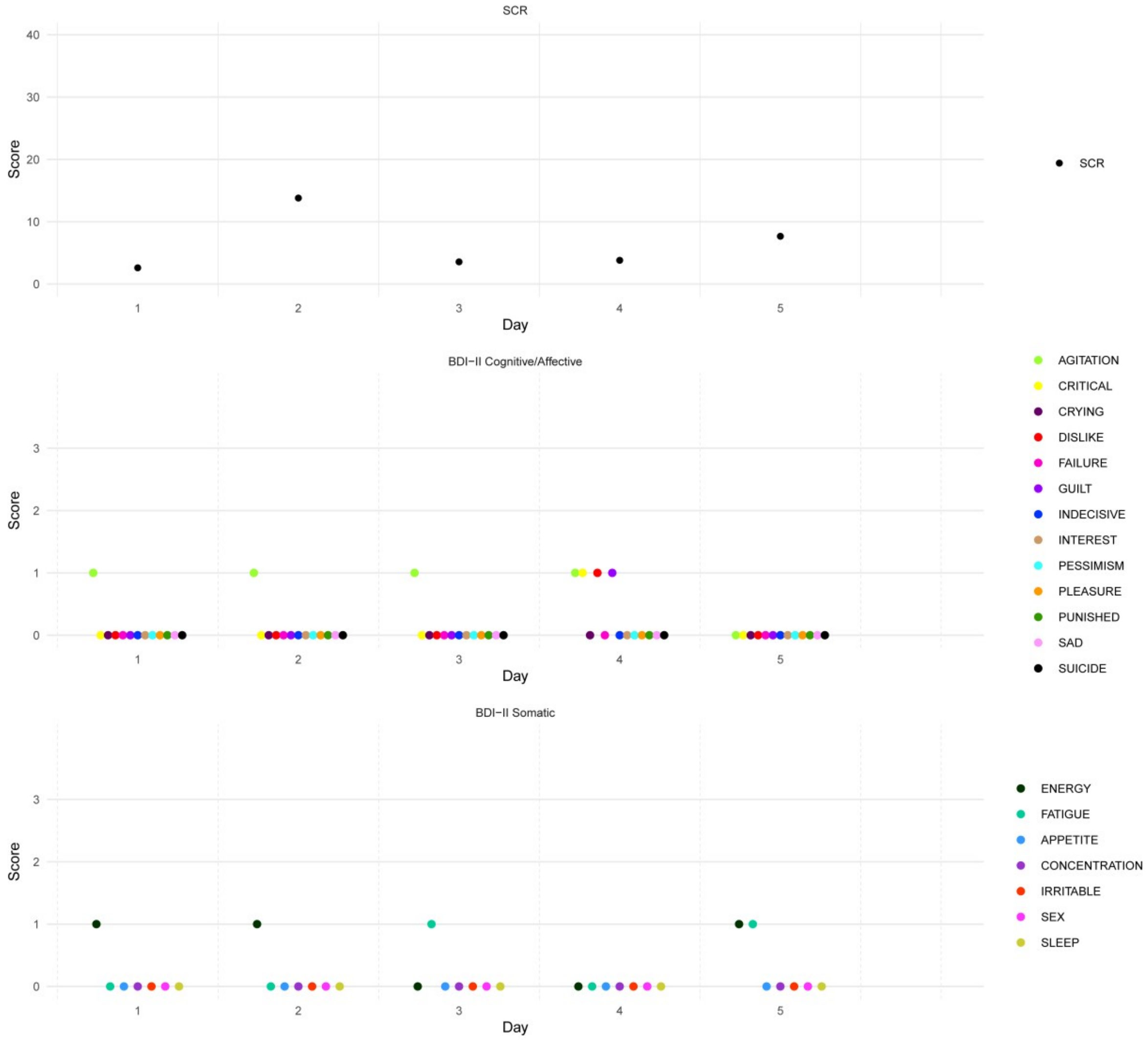

Supplementary Material 7: Participant 8

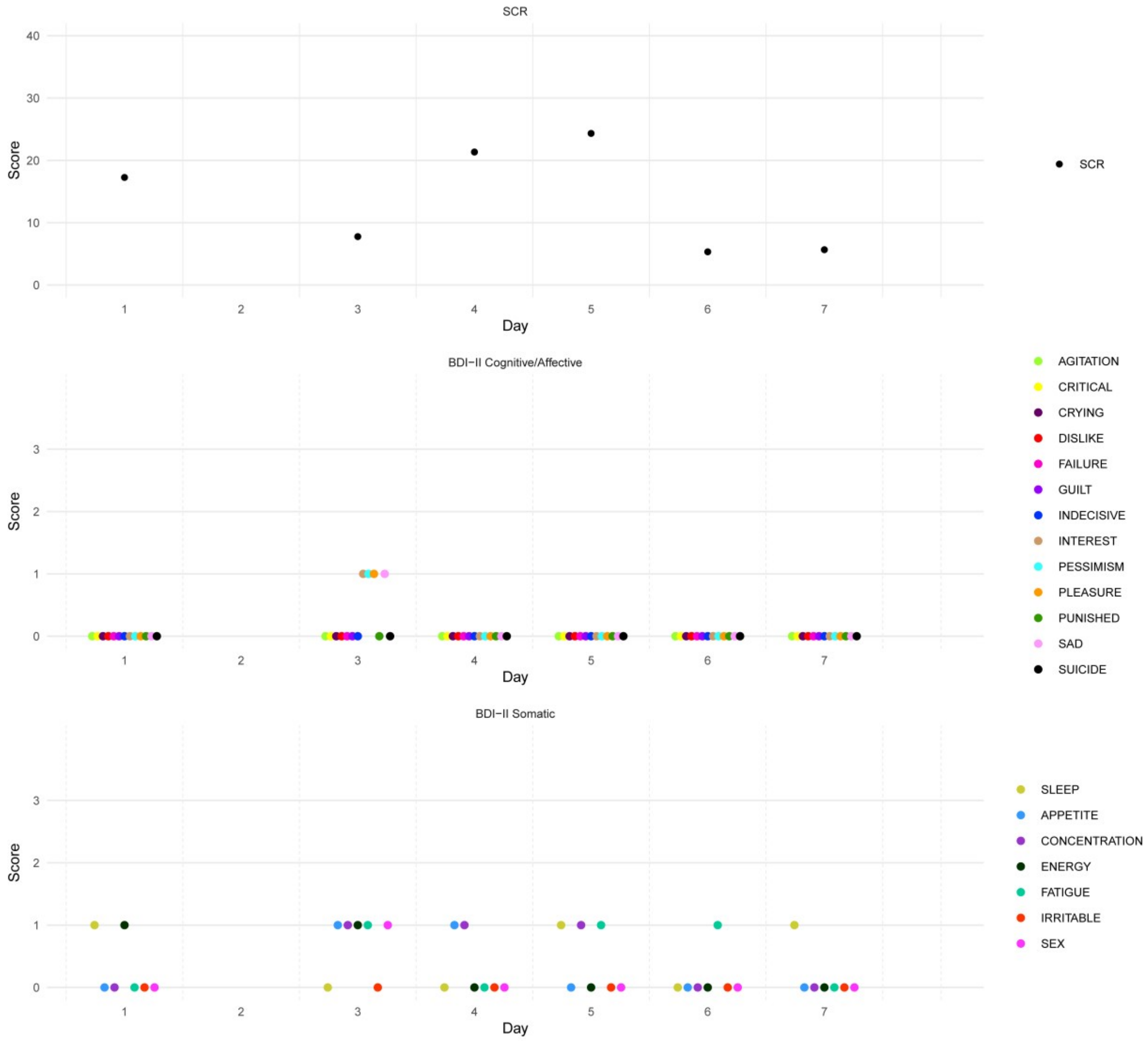

Supplementary Material 8: Participant 9

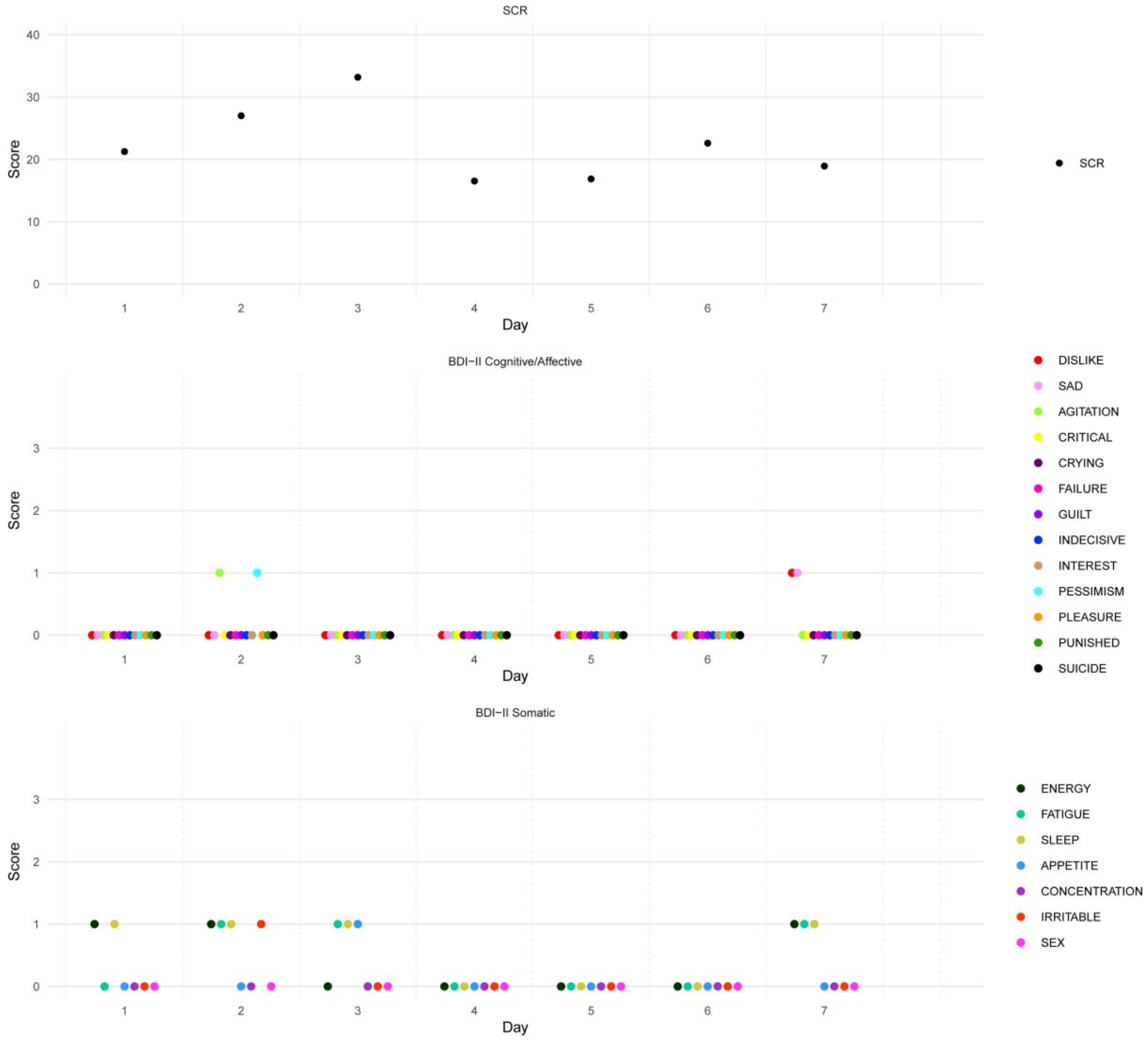

Supplementary Material 9: Participant 10

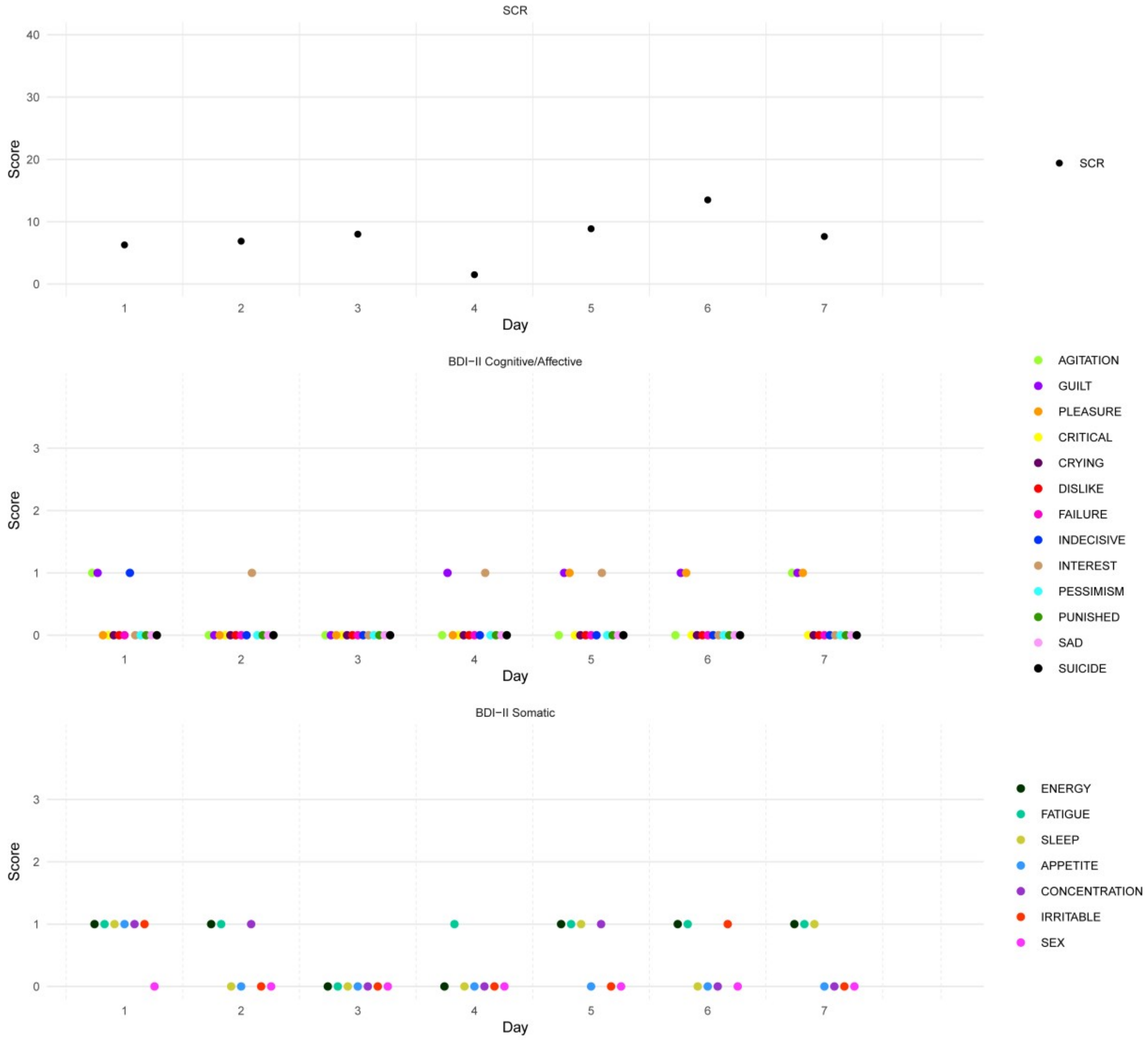

Supplementary Material 10: Participant 11

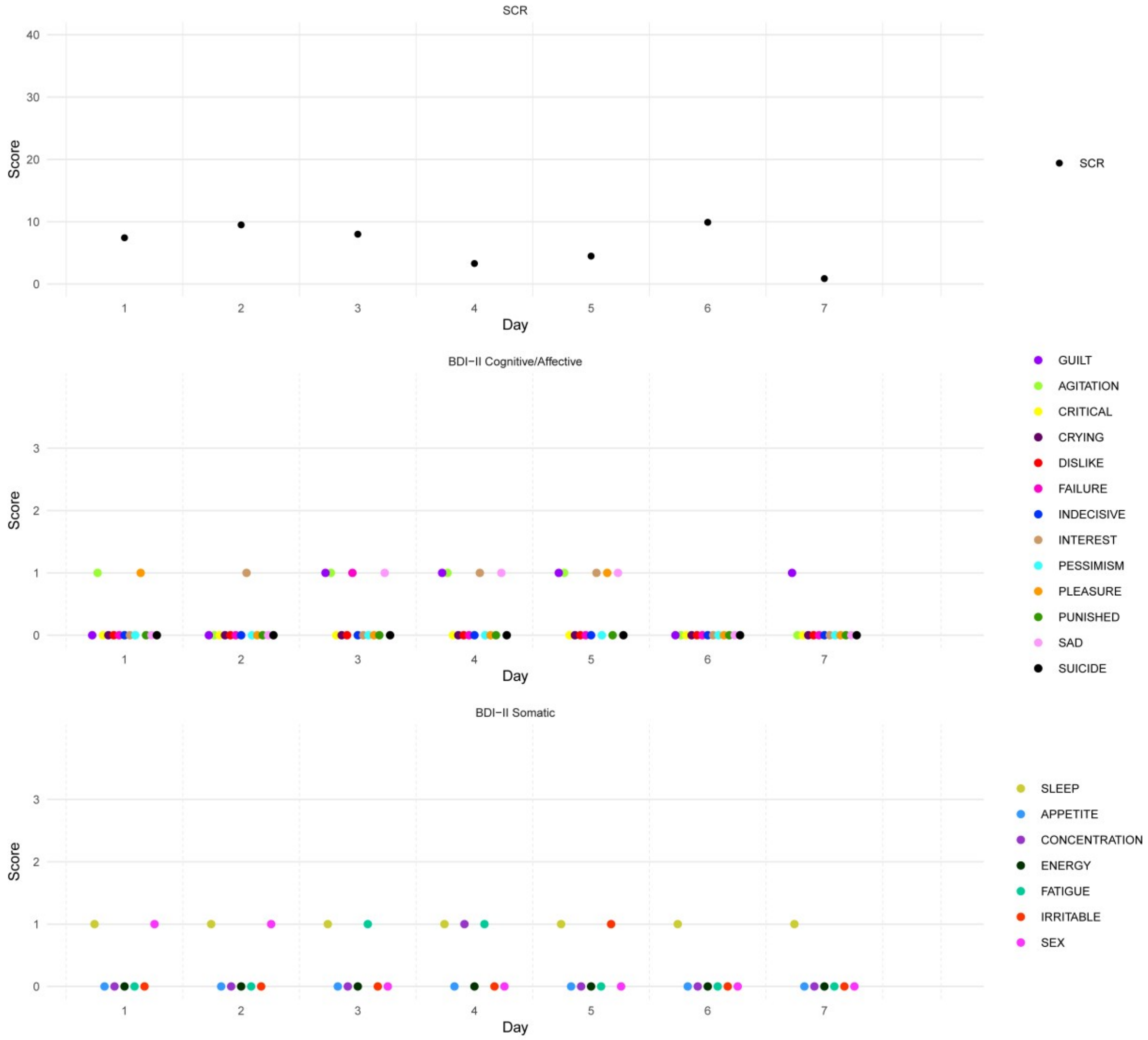

Supplementary Material 11: Participant 12

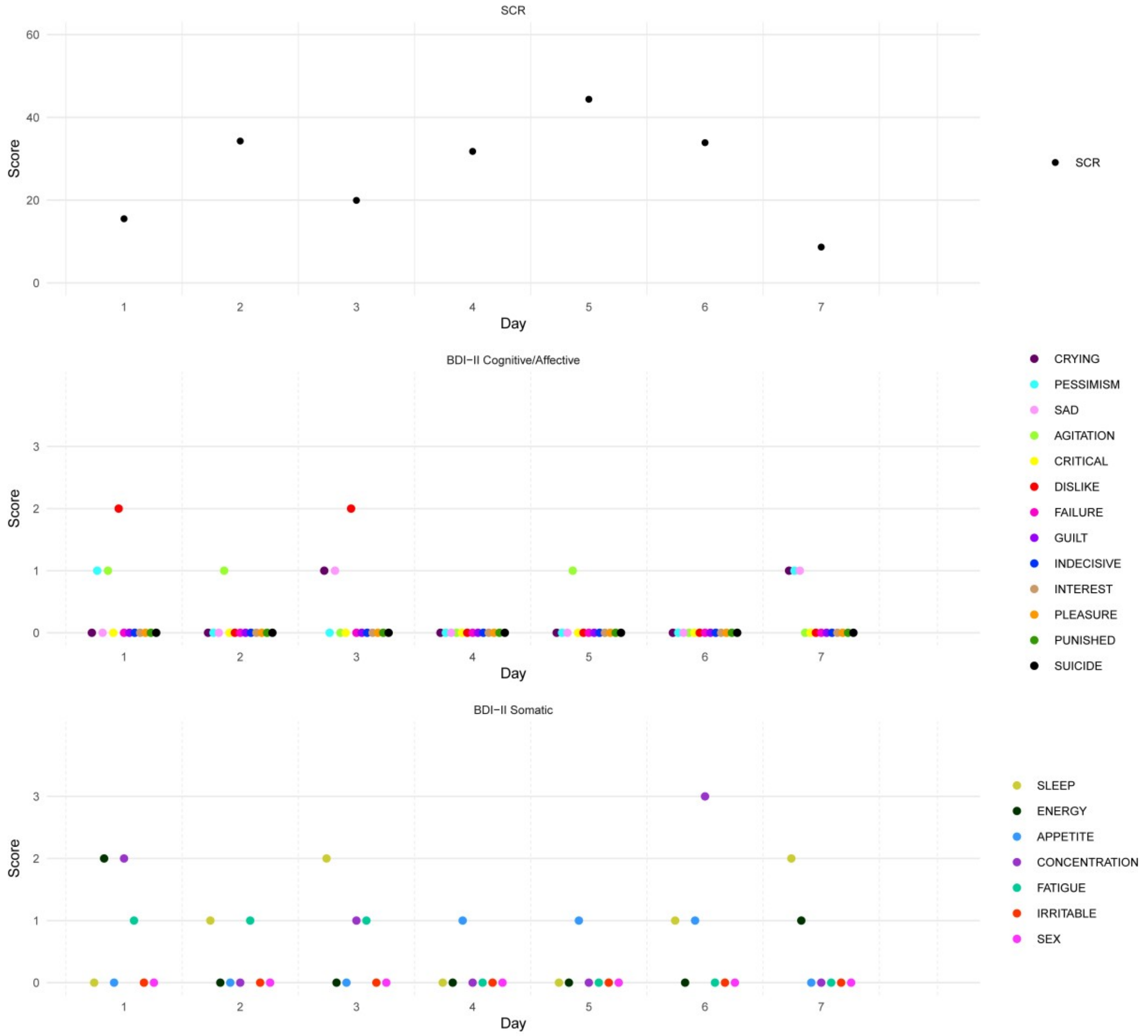

Supplementary Material 12: Chi-square test of association comparing gender distribution of the current pilot study to Abates (2013) systematic review of gender disparity prevalence of depression and Salk et al. (2017) meta-analysis of gender differences in depression.

Abates (2013)

	Female	Male	*Marginal Row Totals*
**Pilot**	10 (7.82) [0.61]	2 (4.18) [1.14]	12
**Population**	63 (65.18) [0.07]	37 (34.82) [0.14]	100
Marginal Column Totals	73	39	112 (Grand Total)

The chi-square statistic is 1.9518. The *p*-value is .162396. *Not* significant at $p < .05$.

The chi-square statistic with Yates correction is 1.1587. The *p*-value is .281739. *Not* significant at $p < .05$.

Salk et al. (2017)

	Female	Male	*Marginal Row Totals*
**Pilot**	10 (8.01) [0.49]	2 (3.99) [0.99]	12
**General population**	195 (196.99) [0.02]	100 (98.01) [0.04]	295
Marginal Column Totals	205	102	307 (Grand Total)

The chi-square statistic is 1.5433. The *p*-value is .214132. *Not* significant at $p < .05$.

The chi-square statistic with Yates correction is 0.8643. The *p*-value is .352539. *Not* significant at $p < .05$.
